# Mild Dermatoglyphic Deviations in Adolescents with Autism Spectrum Disorders and Average Intellectual Abilities as Compared to Typically Developing Boys

**DOI:** 10.1155/2014/968134

**Published:** 2014-11-19

**Authors:** Esther I. de Bruin, John H. Graham, Anneke Louwerse, Anja C. Huizink

**Affiliations:** ^1^Research Institute of Child Development and Education (RICDE), University of Amsterdam, 1018 WS Amsterdam, The Netherlands; ^2^Research Priority Area Yield, University of Amsterdam, 1018 WS Amsterdam, The Netherlands; ^3^Department of Biology, Berry College, Mount Berry, GA 30149, USA; ^4^Department of Child and Adolescent Psychiatry/Psychology, Erasmus MC-Sophia, 3015 CN Rotterdam, The Netherlands; ^5^Department of Developmental Psychology, Faculty of Psychology & Education, VU University Amsterdam, 1081 BT Amsterdam, The Netherlands

## Abstract

Dermatoglyphics, ridge constellations on the hands and feet, are permanently formed by the second trimester of pregnancy. Consequently, they are considered “fossilized” evidence of a specific prenatal period. A high frequency of dermatoglyphic anomalies, or a high rate of dermatoglyphic asymmetry (discordance), is an indication of developmental instability (prenatal disturbances) prior to 24-week gestation. Most dermatoglyphic studies in psychiatry focus on adult schizophrenia. Studies on dermatoglyphic deviances and autism are sparse, include severely disturbed and intellectually retarded patients with autism, and are carried out mainly in non-Western European populations. In this study, finger print patterns, atd-angles, and palmar flexion crease patterns (PFCs) are compared between Western European adolescent teenage males, of average intellect, with Autism Spectrum Disorders (ASD; *n* = 46) and typically developing adolescent teenage males (TD; *n* = 49). Boys with ASD had a higher rate of discordance in their finger print patterns than TD boys. Thus, the hypothesized prenatal disturbances that play a role in the etiology of schizophrenia and severe autism might not be specific to these severe psychiatric disorders but might also be involved in the etiology of varying degrees of ASD.

## 1. Introduction

Dermatoglyphics are the ridge constellations found on the hand palms and foot soles. They have been studied for nearly 120 years [1]. Dermatoglyphics are formed from the 10th week of pregnancy [2] and from the 24th week they remain unchanged and therefore can be considered as fossils of a specific period of prenatal development [3, 4]. The most commonly studied dermatoglyphic indices include finger and palmar ridge counts, palmar atd-angle, ridge patterns on the fingertips, and palmar flexion crease patterns (PFCs). Unusual and atypical dermatoglyphic ridge patterns are often associated with mental disorders such as schizophrenia [5]. This is unsurprising, given the involvement of the nervous system in the timing and development of dermal ridge patterns [6]. Other disorders of neural development, such as autism, which is marked by defective synaptic function and aberrant neural connectivity [7, 8], might also display unusual dermatoglyphic ridge patterns. This study will focus on the association between autism and dermatoglyphic indices.

Dermatoglyphic patterns are highly variable. This phenotypic variation can be subdivided into factorial and stochastic components [9, 10]. The factorial component is the variation among individuals, which can be further subdivided into genetic, macro- and microenvironmental, and ontogenetic components. Dermatoglyphic variation has a strong genetic component (e.g., *h* ^2^ = 0.70 for fingerprint ridge counts, [11]), but it can also be affected by environmental stressors such as infections, pollution, parasites, or the abuse of alcohol or drugs by the mother [12]. The stochastic component is the variation within individuals and it represents developmental instability or developmental noise.

Developmental instability reflects an individual's inability to either correct developmental fluctuations (stability) or to buffer perturbations (resilience). If perturbations are small and constant, one can estimate the stability and resilience of a developmental system by examining its fluctuations [9]. This is usually accomplished by measuring deviations from perfect symmetry of bilateral traits, such as dermatoglyphic traits on left and right sides of the body or brain. There are three types of asymmetry: fluctuating asymmetry, directional asymmetry, and antisymmetry [13, 14]. Of the three, fluctuating asymmetry is regarded as the most relevant when studying associations with mental health outcomes [15]. Fluctuating asymmetry represents random deviations from perfect symmetry between the left and right sides of a bilateral trait [16–20]. These deviations are symmetrically distributed around a mean of zero [9]. Fluctuating asymmetry generally increases as a result of environmental and genetic stress, though the patterns are often inconsistent. In general, it provides an indication of a population's overall state of adaptation and coadaptation [9, 21].

For dermatoglyphic traits, high fluctuating asymmetry can be used to infer prenatal stress and instability, which can have an impact on postnatal development and behavior. Individuals with schizophrenia, for example, have higher dermatoglyphic fluctuating asymmetry [22, 23]. In addition, higher dermatoglyphic asymmetry has been reported for developmentally delayed individuals [24].

In addition to fluctuating asymmetry, dermatoglyphic anomalies have also been used as a marker of developmental instability in psychiatric disorders, mainly adult schizophrenia. In particular, a lowered a-b palmar ridge count has been shown in patients with schizophrenia [4, 25], although this has not been replicated in all studies (e.g., [3, 26]). A meta-analysis showed an overall effect-size (Cohen's *d*) of 0.39 for the reductions of palmar a-b ridge count in schizophrenic patients as compared to unaffected individuals [27]. With respect to finger print patterns, a higher percentage of discordance, as a measure of fluctuating asymmetry, was shown in schizophrenic patients as compared to controls (e.g., [25, 28]). Some other studies, however, failed to find significant differences [3]. With respect to abnormal PFCs, such as the Simian and the Sydney creases, an association with schizophrenia has been shown in different studies (e.g., [26]). Furthermore, deviant PFC patterns, particularly the Simian crease, have been strongly linked with chromosomal anomalies and genetic disorders, such as Down syndrome; the Sydney crease, although rarer, is associated with conditions such as fetal alcohol syndrome and craniofacial syndrome (e.g., [29]).

Mainly older dermatoglyphic studies are available for people with Autism Spectrum Disorders (ASD), disorders of neural development that have a complex genetic basis [30, 31]. ASD in this paper is used as an umbrella term and refers to autistic disorder, Asperger syndrome, and Pervasive Developmental Disorder Not Otherwise Specified (PDD-NOS). Children with ASD were found to have more arches and fewer whorls than typically developing (TD) children [32]. A study of children with intellectual disability (ID) also found more arches, the simplest fingerprint pattern, and more abnormal PFC patterns [33]. Palmar atd-angles are smaller in patients with ASD compared to TD children (e.g., [32]). Not all studies, however, agree on this finding [34]. Arrieta and colleagues [35] found larger atd-angles in girls with ASD than in boys. A few descriptions of dermatoglyphic variables in people with ASD in Eastern European countries (Croatia, Romania, and Serbia) have recently become available. Miličić and colleagues [36] found a smaller atd-angle in patients with ASD as compared to nonaffected TD individuals. Another study showed that boys with ASD had more arches on their fingertips as compared to TD children, as well as a lower total and a-b ridge count and a wider atd-angle [37]. It must be noted, however, that participants in all of these studies were extremely low functioning children with autistic disorder. Although autistic disorder was the primary classification, approximately one-third of the patients in the Eastern European study suffered from profound ID (IQ < 35), whereas more than 50% were diagnosed with mild ID (IQ 35–50). It is therefore unknown whether these dermatoglyphic deviances, as a proxy marker of deviations in early prenatal development, would also show up in children with milder forms of ASD, such as PDD-NOS.

In the current study, we compared (discordance of) fingerprint patterns, atd-angles, and PFC patterns of northern European boys of average intellect and ASD (mainly PDD-NOS), with TD boys. If boys with ASD show, for instance, a higher rate of discordance, this could indicate that disturbances in prenatal development may play a role in the later development of high-functioning ASD.

## 2. Methods

### 2.1. Participants

The 46 male patients (mean age = 16.16, SD = 1.67) were recruited at the Child and Adolescent Psychiatry outpatient department of the Erasmus MC-Sophia, the Netherlands. All patients were diagnosed with a subtype of ASD according to DSM-IV-TR-criteria [38]; more specifically, *n* = 1 Asperger syndrome, *n* = 8 autistic disorder, and *n* = 37 PDD-NOS. Classifications were made by highly ASD-experienced child psychiatrists and validated by administering the Autism Diagnostic Observation Schedule-Generic (ADOS-G; [39]) and the Autism Diagnostic Interview-Revised (ADI-R; [40]). The Diagnostic Interview Schedule for Children (DISC-parent informant; [41]) was used to assess comorbid psychiatric disorders. Of the adolescents diagnosed with ASD, 41% fulfilled diagnostic criteria for an internalizing disorder (i.e., anxiety disorder or depression) and 50% fulfilled criteria for an externalizing disorder (24% met diagnostic criteria for Attention Deficit Hyperactivity Disorder [ADHD], 13% for Oppositional Defiant Disorder [ODD], and 13% fulfilled diagnostic criteria for ADHD and ODD). IQ scores for the total sample (assessed with the Wechsler Abbreviated Scale of Intelligence [42]) were Total Intelligence Quotient (TIQ), M = 103.04 (SD = 14.12), Verbal Intelligence Quotient (VIQ), M = 100.87 (SD = 15.07), and Performance Intelligence Quotient (PIQ), M = 104.57 (SD = 13.45). More specific ASD subtype IQ scores were the following: autistic disorder (*n* = 8), TIQ = 111.50 (SD = 13.07), VIQ = 107.63 (SD = 15.99), and PIQ = 113.50 (SD = 9.21); Asperger syndrome (*n* = 1), TIQ = 108, VIQ = 98, and PIQ = 118; PDD-NOS (*n* = 37), TIQ = 106.63 (SD = 14.83), VIQ = 105.70 (SD = 14.40), and PIQ = 106.23 (SD = 15.70). IQ scores did not differ significantly between groups.

The 49 TD adolescent males (mean age = 15.98, SD = 2.26) were recruited from two different high schools in the Netherlands. Although no IQ tests were administered to the control boys, the level of high school (higher general secondary education and preuniversity secondary education) indicates that IQ scores were most likely above average. To ensure no boys with psychiatric disorders were included in the TD group, the Youth Self Report (YSR) [43] was administered and adolescents who scored above the clinical cutoff point were excluded. The patients and TD adolescent males did not differ in mean age, *t* = 0.47, *df* = 93, *P* = 0.64.

### 2.2. Procedure

**Figure 1:**
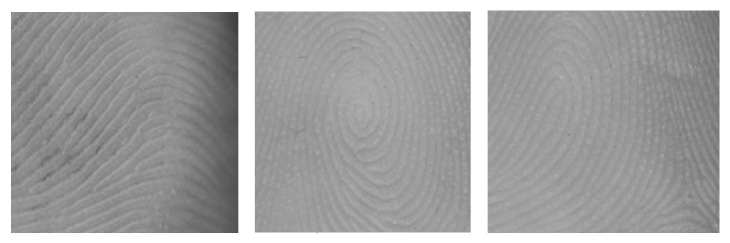
Examples of finger ridge patterns: 1 = arch; 2 = whorl; 3 = loop.

Digital hand scans were made at 1800 dpi with a Hewlett-Packard Scanjet 4600 scanner. Fingerprint patterns were classified according to the three-pattern system (loops, arches, and whorls) [44]. Fingerprint patterns have cores and triradii and the classification is based on the number of triradii present; see also Figure 1. Atd-angles were measured with the Image J software program [45]. PFC patterns were rated as normal or abnormal (Simian or Sydney creases). An interrater reliability (IRR) study was carried out for a subsample of the total sample of *n* = 95. Two independent raters rated a random set of *n* = 28 pairs of hands. Cohen's kappa (*κ*) was 1 for all finger print patterns in all fingers, apart from the little finger of the left hand (for which *κ* = 0.87) and the middle finger of the right hand (for which *κ* = 0.91). Kappa theoretically ranges from 0 to 1 and the values in this study fall within the highest range of agreement according to kappa interpretation guidelines [46]. Further, Spearman's correlations were calculated for the agreement on the continuous variable atd-angle. Correlations were nearly perfect, *r* = 0.96 for the left hand and *r* = 0.98 for the right hand, and thus agreement between raters was extremely high. Moreover, dermatoglyphic analyses were conducted by research assistants who were blind to group membership. Informed consent was obtained prior to participation from parents, caretakers, and adolescents. The Medical Ethics Committee of the Erasmus MC approved the study.

Fingerprint discordance was evaluated finger-by-finger. For example, if an individual's right and left index fingers have different patterns (say loop and arch), they are classified as discordant. If both index fingers have the same pattern, they are classified as concordant. Consequently, fingerprint discordance ranges from 0 (all fingers concordant) to 4 (all fingers discordant, thumb was not included).

### 2.3. Statistical Analyses

Analysis of variance (ANOVA) was used to compare fingerprint patterns, fingerprint discordance, mean atd-angle, and atd-angle asymmetry of boys with ASD and TD boys. Because fingerprint patterns on right and left hands have a Poisson distribution (counts of randomly occurring objects), we used the Anscombe transformation, $2\sqrt{\left(x+3/8\right)}$, to stabilize their variances. Moreover, we only examined whorls and arches because a third variable (loops) is redundant; knowing the numbers of whorls and arches completely determines the number of loops. Previous studies [33, 47] have used contingency tables of fingerprint patterns, but this approach violates the independence assumption of chi-square. The ten fingers of a single individual are not independent observations. The unit of observation is the individual person, not each finger, and this cannot be accommodated in a contingency table analysis. The mean atd-angles (*R* + *L*)/2 were log transformed to stabilize their variances. Unsigned asymmetry of atd-angles was |log⁡_10_⁡*LH*⁡ − log⁡_10_⁡RH|. Cohen's *d* was estimated as a measure of effect size. Chi-square tests were used to assess the association between group (ASD, TD) and PFC pattern (normal, abnormal).

## 3. Results

As can be seen in Table 1, boys with ASD did not differ from TD boys in the number of arches, *F*_1,91_ = 1.44, *P* = 0.23, or whorls, *F*_1,91_ = 0.11, *P* = 0.75. Nevertheless, boys with ASD showed more discordant finger pairs than TD boys, *F*_1,91_ = 6.86, *P* = 0.01. Average atd-angle (log_10_) did not differ between boys with ASD and TD boys, *F*_1,45_ = 1.62, *P* = 0.21. In addition, there was no difference in unsigned asymmetry of atd-angle between the two groups, *F*_1,45_ = 0.99, *P* = 0.33. And no associations were found between group and abnormal PFCs for the left hand, *χ* ^2^ = 0.97, *df* = 1, *P* = 0.33, or the right hand, *χ* ^2^ = 1.96, *df* = 2, *P* = 0.38. In addition, there was no association between PFC discordance and group, *χ* ^2^ = 0.30, *df* = 1, *P* = 0.58. Both TD and boys with ASD showed very low basal rates of abnormal patterns (Simian or Sydney) in their palmar creases. See Table 2 for all PFC information.

## 4. Discussion

This study showed that Western European boys with ASD and average intellect had a significantly higher rate of discordance in their fingerprint pairs than TD boys. They did not differ, however, in the numbers of whorls, the numbers of arches, mean atd-angle, or atd-angle asymmetry. A higher rate of discordance, reflecting greater fluctuating asymmetry, is considered a proxy marker of developmental instability during the second trimester, when the finger ridges form. Thus, the hypothesized altered neurodevelopment that plays a role in schizophrenia and severe autistic disorder might not be specific to these severe psychiatric disorders but might also be involved in the etiology of more broadly defined autism, that is, ASD.

A higher number of arches, as was found in other studies of patients with autism [32, 36, 37], was not found in the boys with ASD in our sample. Arches are the simplest form of fingerprint pattern and seem to be associated with lower intellectual functioning (i.e., [24]). Perhaps this partly explains the (non-)finding in this study. Although the boys in this study were diagnosed with ASD (of which around 80% with PDD-NOS), they were patients with average intellectual abilities (IQ around 100). Patients with ASD in previous studies were profoundly retarded (IQ < 35) and this often coincided with other (i.e., usually chromosomal) disorders. For instance Walker [32] described his patients as suffering from “a profound inability to relate to people, or establish any positive human contact,” which obviously refers to severely disturbed patients, very different from the current study sample.

With respect to the PFCs, no association with ASD was found. This might be related to the extremely low basal rate of the abnormal Simian and Sydney patterns in our sample (i.e., only one Sydney pattern in the right hand was found in the boys with ASD). We know from the literature that these deviant patterns are strongly associated with chromosomal anomalies and genetic disorders, such as Down syndrome [29]. Our sample did not include such patients, which might explain the low basal rate. Further, Sharma and Sharma [48] showed an overview of prevalence of the Simian crease across different populations in the world, and the prevalence in the Netherlands was very low (1.5%) compared to other populations (for instance 14.4% in India).

In summary, a higher rate of discordance, which is an indicator of developmental instability, was found in high-functioning adolescent males with ASD. They showed no other dermatoglyphic deviances. Dermatoglyphic deviances do not seem specific for severely impaired patients with ASD or those with schizophrenia or genetic anomalies (i.e., aneuploidy, deletion, and duplication), but also in the milder forms of ASD (i.e., PDD-NOS), prenatal disturbances might have occurred, which could be reflected in these deviances in the fingerprints. An important note is that to diagnose ASD based on fingerprint asymmetry is not the conclusion that should be drawn from these findings. However, inspection of dermatoglyphics in clinical psychiatric practice could possibly function as an additional observation.

## Figures and Tables

**Table 1:** Fingerprint pattern counts, number of discordant finger pairs, atd-angles, and fluctuating asymmetry of atd-angles for boys with ASD and typically developing boys.

Trait	Character	ASD boys M (SD)	Control boys M (SD)	*P* value	ES (Cohen's *d*)
Fingerprint patterns (Anscombe transformation of counts)	Number of whorls (LH + RH)	2.52 (1.43)	2.42 (1.42)	0.75	0.07
	Number of arches (LH + RH)	1.53 (0.56)	1.74 (1.04)	0.23	−0.25
	Number of discordant finger pairs	2.24 (0.88)	1.80 (0.72)	0.01	0.55

Atd-angles (log_10_⁡ degrees)	(log_10_⁡*LH*⁡ + log_10_⁡ RH)/2	1.61° (0.033°)	1.62° (0.048°)	0.21	−0.38
	|log_10_⁡LH − log_10_⁡ RH|	0.03° (0.05°)	0.02° (0.02°)	0.33	0.30

ASD = Autism Spectrum Disorders; ES = effect size; LH = left hand; M = mean (SD = standard deviation); RH = right hand.

**Table 2:** Palmar flexion creases (PFCs) as a percentage of all palms for boys with ASD and typically developing boys.

Hand	Pattern	ASD boys	Control boys	*χ* ^2^	*P* value
Left	Normal	100% (46)	97.9% (47)	0.97	0.33
	Simian	0% (0)	2.1% (1)		
	Sydney	0% (0)	0% (0)		

Right	Normal	97.8% (45)	93.8% (45)	1.96	0.38
	Simian	0% (0)	4.2% (2)		
	Sydney	2.2% (1)	2.1% (1)		

Discordance between LH and RH		2.2% (1)	4% (2)	0.30	0.58

ASD = Autism Spectrum Disorders; LH = left hand; RH = right hand.

Exact counts of palms are in parentheses. Chi-square tests the hypothesis that there is no association between PFC patterns and group membership.
